# Absorbable implants versus metal implants for the treatment of ankle fractures: A meta-analysis

**DOI:** 10.3892/etm.2013.1017

**Published:** 2013-03-19

**Authors:** ZONG-HUAN LI, AI-XI YU, XIAO-PENG GUO, BAI-WEN QI, MIN ZHOU, WEI-YANG WANG

**Affiliations:** Department of Micro-Orthopedics, Zhongnan Hospital of Wuhan University, Wuhan, Hubei 430071, P.R. China

**Keywords:** absorbable implant, metal implant, ankle fracture, meta-analysis

## Abstract

This meta-analysis was performed to evaluate the efficiency and the safety of absorbable implants. Five major electronic databases (PubMed, Embase, Cochrane Library, SinoMed and Wanfang Data) were systematically searched for randomized controlled trials (RCTs) from their establishment to November 2012. Studies on absorbable implants and metal implants for ankle fractures were selected. The meta-analysis was performed using RevMan 5.1. Ten studies with 762 patients were included and analyzed. Compared with metal implants, absorbable implants used for the internal fixation of ankle fractures produce similar radiographic and functional outcomes (P= 0.52). Normally, removal of the internal fixation is unnecessary (P<0.0001) and the incidence of palpable implants is lower (P=0.02) for absorbable implants. No statistically significant difference was observed between the two groups with regard to foreign body reactions (P=0.07), infection (P= 0.69), osteoarthritis (P= 0.39), pain (P= 0.06), refracture (P=0.67), skin necrosis (P=0.99), deep vein thrombosis (P=0.21) and nerve injury (P=0.94). Absorbable implants used in ankle fractures rarely require reoperation and result in similar functional outcomes and complications compared with metal implants. These characteristics make them efficient and reasonably safe for the treatment of ankle fractures.

## Introduction

Ankle fractures, are the fifth most common type of fracture and account for ∼9.0% of all fractures in the human body, are common worldwide (1). In the United States, it is estimated that ∼260,000 individuals suffer from ankle fractures each year (2). Ankle fractures are most often caused by simple falls, athletic injuries and underlying pathology (2). Every year ∼25% patients with ankle fractures are treated with surgery (3).

Implants, including screws and rods, play an important role in the internal fixation of ankle fractures. Almost all the displaced fractures of the posterior malleolus are fixed with screws and fractures of the medial malleolus are partially fixed (4). Conventional implants made of metal are widely used. However, reoperation is essential to remove the internal fixation, which may cause additional damage to the patients and increase the risk of infection, as well as other complications.

Absorbable implants (AIs) made of polyglycolide (PGA) or polylactide (PLA) have been developed to avoid reoperation (5), which may result in a reduction in costs and psychological benefits. Furthermore, AIs lose their strength gradually and the stress is transferred to the healing bone. Thus, the stress-shielding effect of metal implants (MIs) is reduced. AIs are used for a variety of situations, particularly for joints that are not suitable for repeated surgery, including the reconstruction of the anterior cruciate ligament (ACL) (6) and the fixation of calcaneal fractures (7). The most common use for absorbable materials is displaced ankle fractures (8).

The aim of the current study was to evaluate the efficiency and complications of AIs used for ankle fractures. We consider that this meta-analysis provides strong evidence for the selection of different implants in ankle fractures.

## Materials and methods

### Study design and search strategy

A systematic search of PubMed, Embase, Cochrane Library, SinoMed and Wanfang Data was performed by two authors independently for randomized controlled trials in which metal and AIs are compared for ankle fractures. The search terms used were: ‘absorbable’, ‘bioabsorbable’, ‘biodegradable’, ‘biodegradation’, ‘degradable’, ‘degradation’, ‘polylactide’, ‘polylactic’, ‘polylevolactide’, ‘polylevolactic’, ‘polyglycolide’, ‘polyglycolic’, ‘ankle’, ‘malleolar’ and ‘malleolus’, singly or in combination. When searched in SinoMed and Wanfang Data, related terms were translated into Chinese. There were no limitations on time and publication language.

### Inclusion and exclusion criteria

Studies were included according to the following criteria: i) study design was a randomized controlled trial (RCT), including randomized and quasi-randomized trials; ii) included patients with ankle fractures of all ages; iii) provided comparative information between MIs and AIs for the fixation of the ankle; and iv) no language and time limits set. Studies were excluded when meeting the following criteria: i) studies on ankle fractures with syndesmosis rupture; ii) study designs were case reports, case series, retrospective studies, cohort studies or controlled clinical studies; iii) studies that were redundant or duplicate publications; and iv) studies with <20 patients.

### Data extraction

Data were extracted onto a pre-designed table independently by two reviewers. Then, the tables were exchanged to verify consistency. Discrepancies in outcome extraction were resolved by discussions or a senior reviewer’s opinion. Measurement data and count data in all trials were extracted for meta-analyses, as well as the general characteristics (first author, age, gender, number of patients, study design and intervention) and the descriptive data (average surgery time, length of hospital stay and Olerud and Molander scores) (9).

### Methodological assessment

The methodological quality of the included studies was assessed by the modified Jadad scale (10). During this procedure, eight items, including randomization, blind method, withdrawals, dropouts, inclusion/exclusion criteria, adverse effects and statistical analysis were assessed. The score of the study ranged from 0 (lowest quality) to 8 (highest quality). Studies with scores of 4–8 were considered to be of high quality, while scores of 0–3 were considered poor quality. The strict assessment was performed by one reviewer and verified by the other.

### Outcomes for meta-analysis

The primary outcome measures were excellent and good recovery rate, reoperation, foreign body reaction, infection rate, osteoarthritis and pain. Other complications, including refracture, skin necrosis, deep vein thrombosis (DVT), nerve injury and palpable implants were also assessed. The secondary outcome was a sensitive analysis performed by excluding the studies of low quality (score 0–3).

### Statistical analysis

When the data provided was not appropriate for meta-analysis, the outcome was performed descriptively. Otherwise, the relative risk (RR) and mean difference (MD), with 95% confidence interval (CI), were used as statistical measures to analyze dichotomous variables and continuous data, respectively. Between-study heterogeneity was evaluated using I^2^ statistics. When I^2^>50%, substantial heterogeneity could not be ignored and a random-effects model was adopted. Otherwise a fixed-effects model was used. Statistical analysis was conducted using RevMan 5.1 software for outcome measures. P<0.05 was considered to indicate a statistically significant difference.

## Results

### Identification of relevant literature

**Figure 1:**
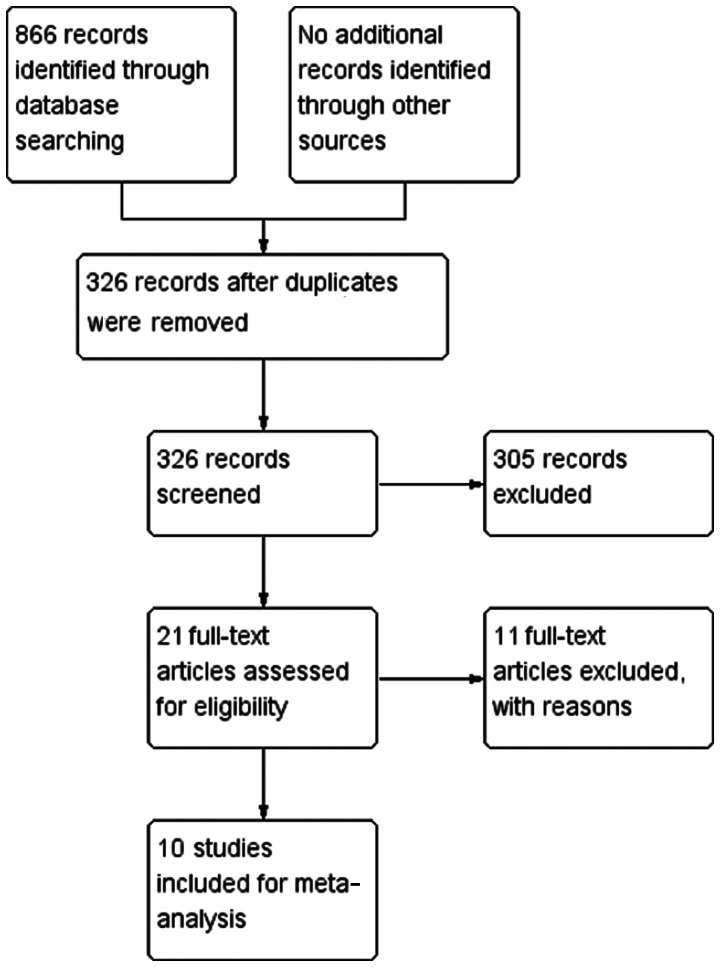
Flow diagram of the study screening.

A flow diagram and results of the literature screening are presented in Fig. 1. The final review included 10 RCTs (11–20) with a total of 762 patients. The general characteristics of the 10 included studies are summarized in Table I.

### Methodological quality assessment

The scores of the study are presented in Table I. The majority of the studies had a score of 4–6 (16), indicating that they are of high quality, while three studies were of low quality with a score of 3 (19) or 2 (13,14). Randomization was described in all studies. However, two (11,18) were stated to have used a sealed envelope system. In one of the two quasi-RCTs, the patients were randomized by the date of the injury (17), while in the other, they were randomized by registration order (12). None of the studies used the blind method. For the measurements that were descriptive, statistical analysis was described only in three studies (11,12,18).

### Radiological assessment

Radiological outcomes, including the redisplacement of fractures, were mentioned in the majority of studies. In one of the studies, judgments made from radiographs indicated that the redisplacements in the two groups were similar in number as well as extent (18). By contrast, patients in the study by Kankare *et al*(16) suffered smaller redisplacements with a similar number in the two groups. Contrastingly, another study by Kankare *et al*(17) demonstrated that 8/16 patients in the PGA group and 1/13 patients in the Arbeitsgemeinschaft für Osteosynthesefragen (Association for the Study of Internal Fixation, ASIF) (AO) group had redisplacements. However, these were not due to iatrogenic reasons, but due to poor bad compliance following treatment. The results from the study by Dijkema *et al*(19) revealed that all fractures healed without any displacement, which further confirmed the effect of compliance.

### Functional assessment

**Figure 2:**
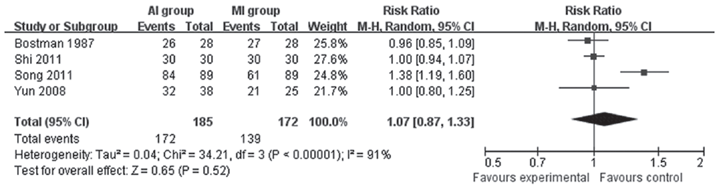
Excellent and good recovery rate in the ankle functional evaluation in the AI and MI groups. AI, absorbable implant; MI, metal implant; CI, confidence interval.

Functional outcomes, including Olerud and Molander functional score, excellent and good recovery rate, range of motion and return to preoperative level, are mentioned in these studies. Five studies (15–18,20) compared the Olerud and Molander scores with different implants. The scores in the AI group tend to be higher than those of the MI group; however, there was no statistically significant difference. Functional outcomes were evaluated by an excellent and good recovery rate in four studies (12–14,20). As shown in Fig. 2, no significant difference was detected.

Following surgery, the range of ankle motion is significantly reduced; however, as indicated in one study (15), the reduction has no correlation with the type of the screw used. This is in disagreement with the study by Kankare *et al*(16), which observed that 2/16 patients in the self-reinforced polyglycolide (SR-PGA) group and 8/19 patients in the metal implant group had limited motion. Another study (17) also stated that 3 patients with AO implants had limited motion. Thus, no consensus was established based on the current evidence.

The study by Bucholz *et al*(18) demonstrated that the majority of patients in the two groups returned to pre-injury work status. No significant difference was detected in the two groups in the ability to walk, run, jump and climb stairs. Outcomes from Kankare *et al*(17) revealed that one patient in the PGA group lost dorsiflexion. Differences were not statistically significant among all these studies.

### Meta-analysis

**Figure 3:**
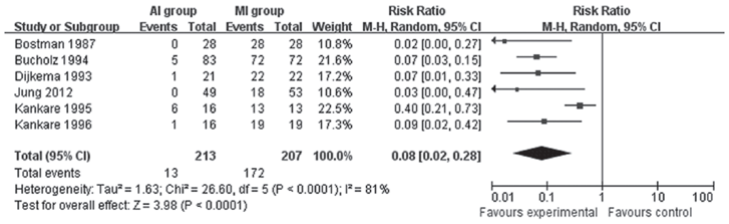
Incidence of reoperation in the AI and MI groups. AI, absorbable implant; MI, metal implant; CI, confidence interval.

The excellent and good functional recovery rate in the AI and MI groups were compared in four studies (12–14,20) and were observed to be similar (RR=1.07; 95% CI= 0.87–1.33; P= 0.52; Fig. 2). The number of patients that required reoperation was counted in six studies (11,16–20). The AI group required significantly fewer reoperations compared with the MI group (RR=0.08; 95% CI=0.02–0.28; P<0.0001; Fig. 3).

**Figure 4:**
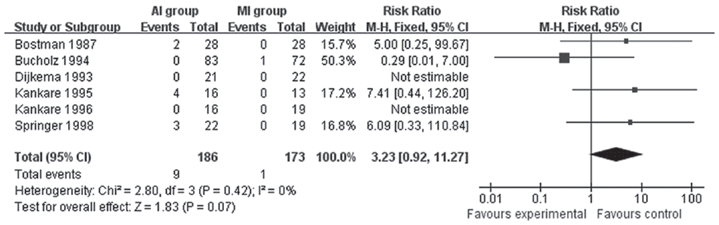
Incidence of foreign body reaction in the AI and MI groups. AI, absorbable implant; MI, metal implant; CI, confidence interval.

**Figure 5:**
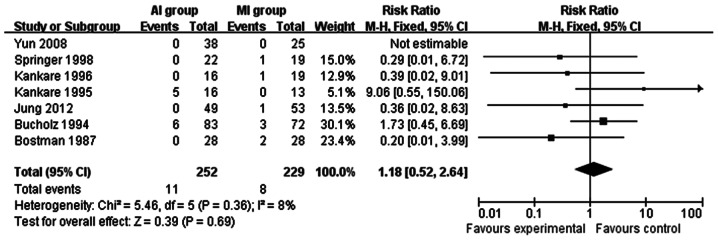
Incidence of infection in the AI and MI groups. AI, absorbable implant; MI, metal implant; CI, confidence interval.

**Figure 6:**
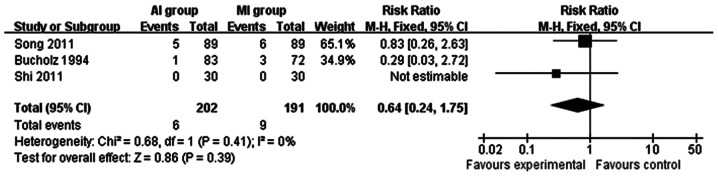
Incidence of osteoarthritis in the AI and MI groups. AI, absorbable implant; MI, metal implant; CI, confidence interval.

**Figure 7:**
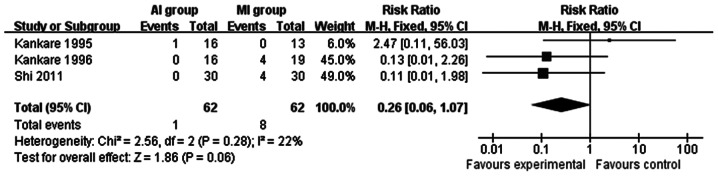
Incidence of pain in the AI and MI groups. AI, absorbable implant; MI, metal implant; CI, confidence interval.

Complications following surgery were mentioned in all included studies. Foreign body reactions were compared in six studies (14–19). Patients treated with absorbable materials are more likely to suffer sterile effusion, sinus formation and osteolysis (RR=3.23; 95% CI=0.92–11.27; P=0.07; Fig. 4). Seven studies (11,14–18,20) compared the infection rate between the MI group and the AI group. The results revealed that there was no statistically significant difference between the two groups (RR=1.18; 95% CI=0.52–2.64; P=0.69; Fig. 5). Osteoarthritis, recorded in three studies (12,13,18), was not significantly different (RR= 0.64; 95% CI= 0.24–1.75; P= 0.39; Fig. 6). Patients treated with AIs had improved results with regard to the incidence of pain (RR=0.26; 95% CI=0.06–1.07; P=0.06; Fig. 7). Furthermore, as shown in Table II, no significant differences were detected with regard to refracture (RR=0.68; 95% CI=0.12–3.92; P=0.67), skin necrosis or sloughs (RR=1.01; 95% CI=0.15–6.92; P=0.99), DVT (RR= 0.31; 95% CI= 0.05–1.91; P=0.21) and nerve injury (RR=0.94; 95% CI=0.19–4.74; P=0.94); however, a significant difference was observed in the incidence of palpable implants (RR=0.68; 95% CI=0.50–0.93; P= 0.02).

### Sensitivity analysis

Sensitivity analysis was conducted by excluding the studies (13,14,19) of low quality (scores 0–3). The study data did not change with respect to the outcomes of infection, skin necrosis, DVT and nerve injury following exclusion, respectively, and there was only one study with respect to refracture. Thus, sensitivity analyses of these outcomes were not performed. I^2^, RR and 95% CI of all outcomes are shown in Table III. The results suggest that all of the excluded studies had no bias on the outcomes and the results reported in this study are acceptable.

## Discussion

The results of our study revealed that the functional outcomes were not significantly different between the AI and MI groups. Reoperation was seldom necessary for ankle fractures fixed with AIs, unless refractures occurred, implants broke or the local response was serious. This benefits the patients financially and physiologically. However, aside from the incidence of palpable implants, the rate of foreign body reaction, infection, osteoarthritis, pain, refracture, skin necrosis, DVT and nerve injury were similar in the two groups.

The incidence of ankle fractures is gender-related (21). For males the peak age range is 15–24 years, whereas it is 65–75 years for females. This is in accordance with the results in our study (data not shown). In the study by Shi *et al*(13), the gender ratio was 49/11 (male/female) with an average age of 36 years, while another study (16) with 9 males and 26 females had an average age of 72/73 years (absorbable/metal).

When treated with different implants, the excellent and good recovery rates were not significantly different in the ankle functional evaluation (Fig. 2). Rangdal *et al* conducted a prospective study (22) to assess the functional recovery of ankle fractures treated with AIs. With plaster immobilization and no bearing of weight, the results were satisfactory. However, before concluding that AIs are similar or even slightly better than metal ones in function, the heterogeneity, study design and the number of patients included should not be ignored.

The lower reoperation rate of the AI group compared with the MI group may be due to the biodegradable and absorbable charactistics of the implant used *in vivo* without removal. Yet, there were several patients that required reoperations for the following two reasons: i) the non-specific tissue response elicited by the degradation and absorption of the materials in the tissue and ii) AIs made of polymer materials are not as strong as metal ones. Refracture is a great threat following surgery (23). As Kankare *et al*(17) declared, certain patients did not follow the post-operative instructions and their screws broke, which required immediate surgical removal.

Absorbable materials made of PGA or PLA are broken down via hydrolysis in the body (24). With the accumulation of the breakdown products, foreign body reactions, including sterile effusion, sinus formation and osteolysis around the implants, are triggered (25). The results indicated that foreign body reactions are more likely to occur with absorbable materials, although no significant difference was detected. The incidence of foreign body reactions in our study was 4.8% (9/186), which is slightly lower than that of 6.1% reported in the study by Böstman (26). Although foreign body reactions occurred and fluid accumulated topically, the fracture healing was uneventful (27). AIs had no specific inhibitory and stimulatory effects on bone compared with metal materials (28).

The difference in the incidence of infection is not statistically significant. However, patients in the AI group had a tendency of reduced infection compared with those in the MI group. This may be due to the small sample size. A study (29) with a large sample size compared the infection rate between AIs and metal fixation and detected no significant difference. Theoretically, the degradation of the polymer results in the accumulation of sterile effusion, which leads to topical susceptibility to infection. However, the risk of infection may increase along with incidence of reoperation in the MI group. Therefore, it is doubtful whether a significant difference would be detected if the sample size was enlarged.

There were no statistical differences in refracture, skin necrosis, DVT, nerve injury, palpable implants and pain, presented in Table II and Fig. VII, between the two groups. We consider that this is due to the following reasons: i) no difference exists substantially; ii) the weakness of the included studies, including the small sample size, large number of patients lost to follow-up and the incompleteness of measurements; and iii) these complications are nonspecific and are affected by a number of factors, including the types of fractures and the body mass index (BMI) (30). However, the incidence of palpable implants was higher in the MI group. To a certain degree, the biodegradable property of AIs accounts for this. Another cause may be that the determination of palpable implants is subjective.

As shown in Table III, the results of the sensitivity analyses indicate that the meta-analysis results are stable and accurate. Following exclusion of an article, although the results for palpable implants were determined to not be significantly different, this may be explained by the sample size allowing for the existence of a clear tendency. Furthermore, following exclusion of the three studies, sensitivity analyses caused slight changes in the results; however, they were not conclusive.

Heterogeneity must be noted as a weakness of our study. Clinical heterogeneity may exist objectively owing to the complexity of ankle fractures, distribution of age, subjective evaluation and heterogeneity of treatments. Once the patients were admitted, treatments may have differed due to variations in the specific degree of injury, age and the willingness of the patients, despite the use of the same surgeon. There was a high statistical heterogeneity (I^2^=81%) for the results of reoperation (Fig. 3). By excluding a study of low quality, the heterogeneity was even higher (I^2^=85%). Following exclusion of the other article (16), the heterogeneity decreased dramatically (I^2^= 0%) with an extremely slight change in the result (RR=0.06; 95% CI=0.03–0.12; P<0.00001). This may be explained by the poor compliance of the patients included. AIs are not as strong as MIs and are prone to breakage. Thus, compliance and post-operative nursing are important for the uneventful recovery of patients.

Although the results of our study are based on the best evidence currently available, there remain limitations that need to be addressed. Firstly, the total number of cases is small, which may be a possible reason for the lack of a significant difference. Secondly, the follow-up times, the majority of which were 12 months or less, were relatively short. The time until the occurrence of adverse tissue reactions to PGA and PLA was 11 weeks and 4.3 years, respectively, following surgery (31). Certain chronic complications, including post-traumatic osteoarthritis with a latency time of 20.9 years (32), would not be detected. Although osteoarthritis was reported in a study (18), it was likely to be incomplete. The relatively large number of patients lost to follow-up may also affect the validity of the study. Furthermore, all studies were conducted in different places without a blinding method. This determined the differences in the incompleteness of the reported results and the inconsistency of the scoring criteria, particularly the subjective ones. In addition, several studies described the statistical methods; however, only means without standard deviations were provided in a number of cases with quantitative data, particularly the information on functional measurements. Thus, these data were analyzed descriptively without meta-analysis. Finally, the majority of the articles included in our review are relatively old. We expanded the research; however, this produced only reviews and case series that did not meet the inclusion criteria.

There has been a wide range of applications for AIs in orthopedic use, including reconstruction of the ACL (7), fixation of type II odontoid fracture (33) and even maxillofacial surgery (34), which are not weight bearing. However, the ankle joint is weight bearing. In the view of the majority of doctors, the fixation of implants is not strong enough to secure and stabilize the ankle. Thus, the risk of refracture may increase. In consideration of this, patients were immobilized with plaster for six weeks routinely and weight bearing was allowed gradually following surgery. Therefore, the activities of the ankle and the weight bearing of implants and the ankle were reduced to a certain extent, which reduced the rate of refracture. However, immobilization also leads to poor circulation and topical hemodynamic changes. The potential risk of DVT increases. Among all relevant studies, the incidence rate of DVT was low and similar in the two groups. This may be a false finding, caused by the small sample size and the weakness of the design. Therefore, large strictly designed and high-quality multi-center, randomized, double-blinded studies are required to confirm the results of the current study.

AIs for the treatment of ankle fractures do not typically require reoperation and result in similar functional outcomes, as well as complications, compared with MIs. These implants are safe and efficient enough for the management of ankle fractures. More high-quality, larger scale, randomized controlled trials are required to confirm this conclusion.

## Figures and Tables

**Table I:** General characteristics of included studies.

Study	Location	Cases	Average age (years)	Study design	Randomized method	Intervention	Comparison	Jadad score
Jung 2012	Korea	53/56	>16	RCT	Sealed envelope	FreedomPlate, screw	Metallic plate, screw	6
Song 2011	China	89/89	18–68	Quasi RCT	Registration order	Absorbable screw	Titanium alloy screw	4
Shi 2011	China	30/30	36 (17–55)	RCT		Absorbable screw	Metal screw	2
Yun 2008	China	38/25	16–76	RCT		Absorbable screw	Metal screw	2
Springer 1998	Netherlands	22/19	16–75	RCT		Biofix implant	Standard AO fixation	4
Kankare 1996	Finland	16/19	72 (65–86)/73 (65–90)	RCT		PGA implant	Metallic implant	4
Kankare 1995	Finland	16/13	46 (29–68)/46 (31–62)	RCT	Sealed envelope	PLA screw	Stainless steel screw	5
Bucholz 1994	America	83/72	40/39	Quasi RCT	Date (odd or even)	PGA screw; PLLA screw	AO implant	4
Dijkema 1993	Netherlands	21/22	16–70	RCT		Biofix implant	Metal implant	3
Bostman 1987	Finland	28/28	38.3/41.6	RCT		Biodegradable implant	Metal implant	4

-/-, no. in the absorbable implant group/no. in the metal implant group; RCT, random controlled trial; PLA, polylactic acid; PLLA, polylevolactic acid; PGA, polyglycolide acid; AO, Arbeitsgemeinschaft für Osteosynthesefragen.

**Table II:** Meta-analysis of selected outcomes.

	All included studies				
Outcomes	No.	Cases	I^2^ (%)	RR (95% CI)	P-values
Refracture	2	99	35	0.68 (0.12–3.92)	0.67
Skin necrosis	2	190	0	1.01 (0.15–6.92)	0.99
DVT	3	105	0	0.31 (0.05–1.91)	0.21
Nerve injury	3	219	0	0.94 (0.19–4.74)	0.94
Palpable implant	4	352	0	0.68 (0.50–0.93)	0.02

RR, relative risk; CI, confidence interval; DVT, deep vein thrombosis.

**Table III:** Sensitivity analysis.

	All included studies					Studies of high quality				
	
Outcomes	No.	Cases	I^2^ (%)	RR (95% CI)	P-value	No.	Cases	I^2^ (%)	RR (95%CI)	P-value
Excellent and good rate	4	357	91	1.07 (0.87–1.33)	0.52	2	234	95	1.15 (0.76–1.74)	0.51
Reoperation	6	420	81	0.08 (0.02–0.28)	<0.0001	5	377	85	0.08 (0.02–0.36)	0.0008
Foreign body reaction	6	359	0	3.23 (0.92–11.27)	0.07	5	316	0	3.23 (0.92–11.27)	0.07
Osteoarthritis	3	393	0	0.64 (0.24–1.75)	0.39	2	333	0	0.64 (0.24–1.75)	0.39
Pain	3	124	22	0.26 (0.06–1.07)	0.06	2	64	47	0.40 (0.07–2.20)	0.29
Palpable implant	4	352	0	0.68 (0.50–0.93)	0.02	3	292	0	0.73 (0.53–1.00)	0.05

RR, relative risk; CI, confidence interval.
